# The Perrault Syndrome Mystery: A Case Report on Its Diagnosis in a 26-Year-Old Female

**DOI:** 10.7759/cureus.70648

**Published:** 2024-10-01

**Authors:** Mahwish Iqbal, Ayesha Jamal, Ruqayyah A Ahmed

**Affiliations:** 1 Department of Obstetrics and Gynecology, Naseem Jeddah Medical Center, Jeddah, SAU; 2 Department of General Medicine and Surgery, Batterjee Medical College for Science and Technology, Jeddah, SAU

**Keywords:** gonadal dysgenesis, karyotype, ovarian failure, perrault syndrome, sensorineural hearing loss

## Abstract

Perrault syndrome (PRLTS) is a rare autosomal recessive disorder characterized by sensorineural hearing loss in both sexes and ovarian dysfunction in females with a 46, XX karyotype. Due to its rarity and diagnostic challenges, herein we report on a 26-year-old woman who presented with secondary amenorrhea, congenital deafness in one ear, and progressive hearing loss in the other. Physical examination showed poorly developed breasts and normal external genitalia. Lab tests revealed high follicle-stimulating hormone (FSH) levels, indicating ovarian failure. Imaging revealed a small uterus and streak ovaries without follicular activity. Initially misdiagnosed with various overlapping syndromes such as Turner, Turner mosaic, and Swyer syndromes, she was started on oral contraceptive pills which induced menstruation and minimal breast development but caused mood swings and depression, leading to inconsistent use. Later, karyotyping revealed a normal 46,XX karyotype, shrouding the case in mystery. A few years later, after additional investigations, her hearing loss and reproductive disruptions were connected, and she was diagnosed with PRLTS. The absence of neurological symptoms suggests type I PRLTS. This case underscores the diagnostic challenges of PRLTS and highlights the importance of genetic testing for accurate diagnosis. It also emphasizes the need for a multidisciplinary approach and further research to improve understanding and management of this rare condition.

## Introduction

Perrault syndrome (PRLTS) is an extremely rare autosomal recessive disorder affecting both males and females [1]. It is characterized by two important features: sensorineural hearing loss in both genders, and primary amenorrhea and ovarian dysfunction in females with a 46, XX karyotype [2]. Biallelic pathogenic mutations in one of the six genes (CLPP, ERAL1, HARS2, HSD17B4, LARS2, or TWNK) are required for the diagnosis; however, in about 60% of cases of PRLTS that have been documented so far, a molecular diagnosis cannot be made [3]. Less than thirty cases of PRLTS have been documented in the medical literature, and research into this rare disease is still ongoing. Herein we report an unusual case, which, after a series of investigations, was diagnosed as PRLTS.

## Case presentation

A 26-year-old unmarried female presented to the OPD with the chief complaint of secondary amenorrhea. She had been deaf in her right ear since birth and suffered from progressive hearing loss in her left ear. She was noted to be wearing a hearing aid in her left ear. She had also been experiencing alopecia for many years.

She was born to parents of a non-consanguineous marriage. She is the youngest child in her family. She has two sisters and one brother, all of whom are normal and healthy, with normal hearing and age-appropriate development of secondary sexual characteristics. One of her sisters is married and has a daughter. There was no history of similar complaints or any other significant medical conditions in the family. Both her mother and sisters began menstruation at age 13.

Upon physical examination, the patient had poorly developed breasts and developed pubic and axillary hair corresponding to Tanner stage III. Her height was 174 cm and weight was 70 kg. Her external genitalia were normal. Her vital signs were within the normal range. The systemic evaluation came out normal.

The patient had no abnormal neurologic symptoms, with normal gait and vision. She has normal intelligence and is able to perform her daily tasks well. She works in a bank in the information management department.

**Figure 1:**
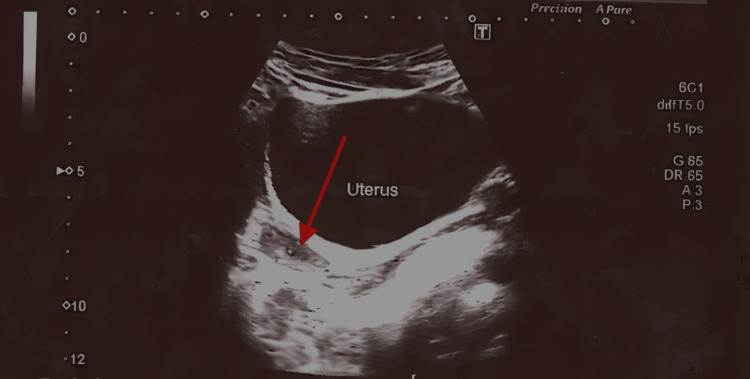
Ultrasound of the pelvis showing an anteverted, small uterus. The red arrow indicates the uterus.

**Figure 2:**
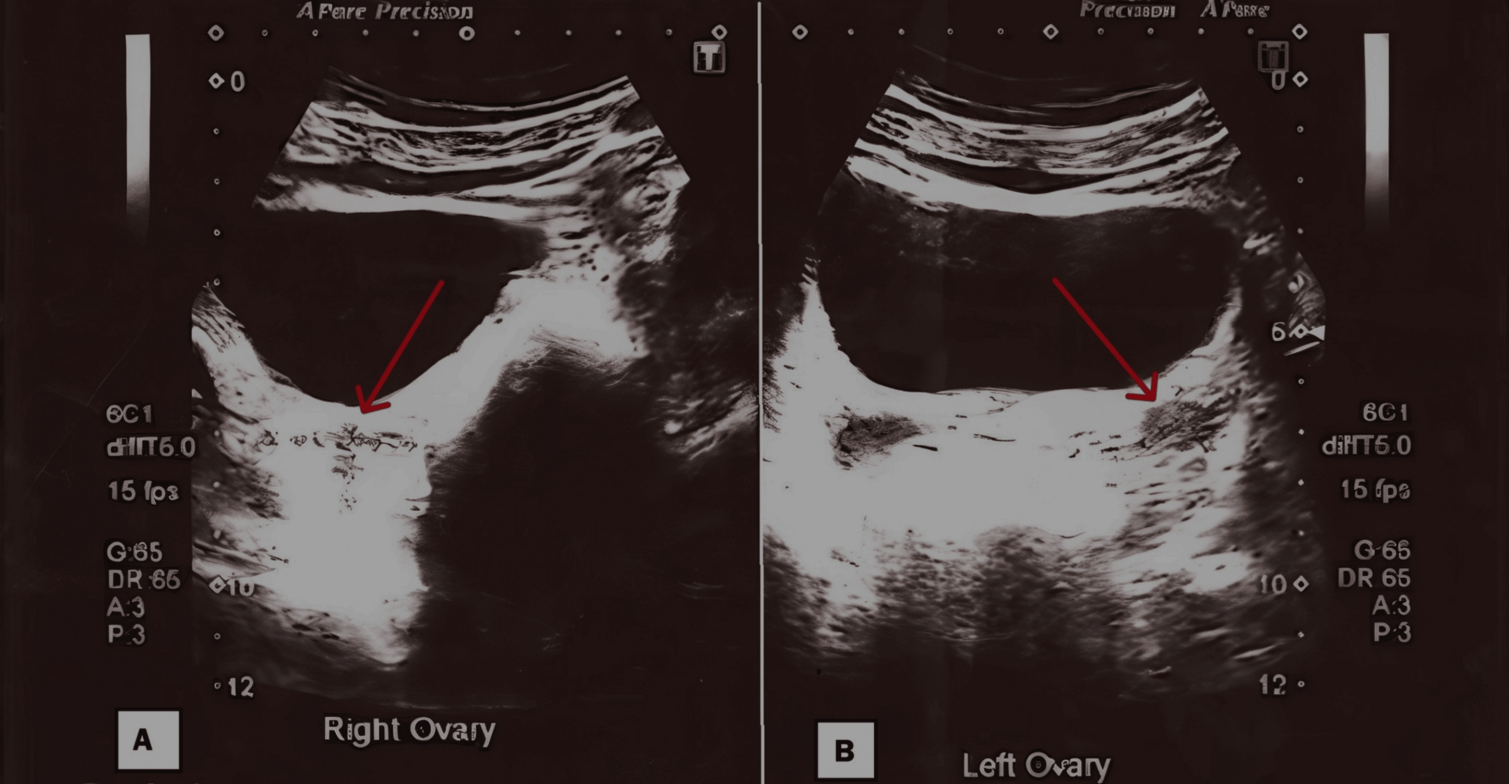
Ultrasound of the pelvis showing streak ovaries (A, B). Red arrows point towards the streak ovaries. A: Right Ovary, B: Left Ovary.

Radiological investigation included an ultrasonogram of the abdomen and pelvis, which showed an anteverted and small uterus measuring 6.0 x 2.3 x 1.5 cm, with a relatively bulky cervix compared to the body. No fibroid was seen. The endometrium was central and 4.00 mm thick (Figure 1). Ovaries were small and measured to be 2.2 x 1.0 cm (right) and 1.9 x 1.2 cm (left). No follicular activity, cystic or solid lesion was noted (Figure 2). No adnexal masses were seen.

**Figure 3:**
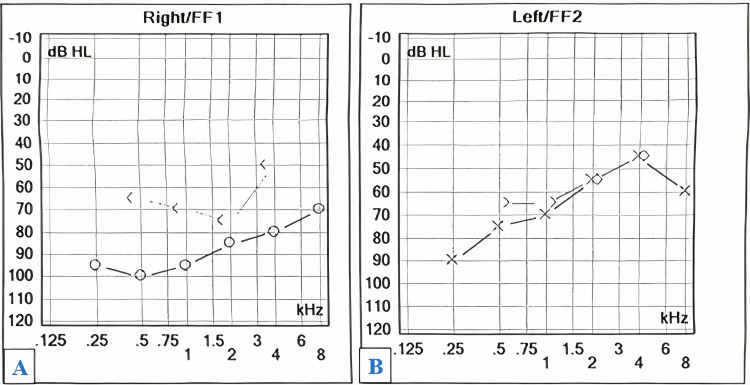
Audiogram showing sensorineural hearing loss in both ears (A, B). A: Severe sensorineural hearing loss in the right ear. B: Moderate to severe sensorineural hearing loss in the left ear. Graph key: X: Air conduction threshold for the left ear. O: Air conduction threshold for the right ear. *>:* Bone conduction threshold for the left ear. *<:* Bone conduction threshold for the right ear.

Audiometric evaluation suggested severe sensorineural hearing loss in the right ear and moderate to severe sensorineural hearing loss in the left ear (Figure 3). The patient had undergone hormonal studies, at the age of 17, which revealed that her follicle-stimulating hormone (FSH) levels were very high and in the menopausal range (Table 1).

**Table 1:** Endocrinology lab result.

Hormone	Value	Phase reference	Range	Remarks
Follicle stimulating hormone (FSH)	38.9 IU/I	Follicular phase	2.2-15.0	FSH is high and in menopausal range
		Preovulatory phase	2.6-100.0	
		Luteal phase	1.3-10.0	
		Postmenopausal	27.0-129.0	
Luteinizing hormone (LH)	17.8 IU/I	Follicular phase	0.8-27.1	LH is high and in menopausal range
		Preovulatory phase	9.6-155.0	
		Luteal phase	0.7-24.5	
		Postmenopausal	13.5- 96.0	
Prolactin (PRL)	14.2 ng/ml	Cyclic female	1.0-27.0	PRL is normal
		Post-menopausal	2.0-13.0	
Thyroid stimulating hormone (TSH)	1.586 mIU/L		0.34–4.25	TSH is normal

**Figure 4:**
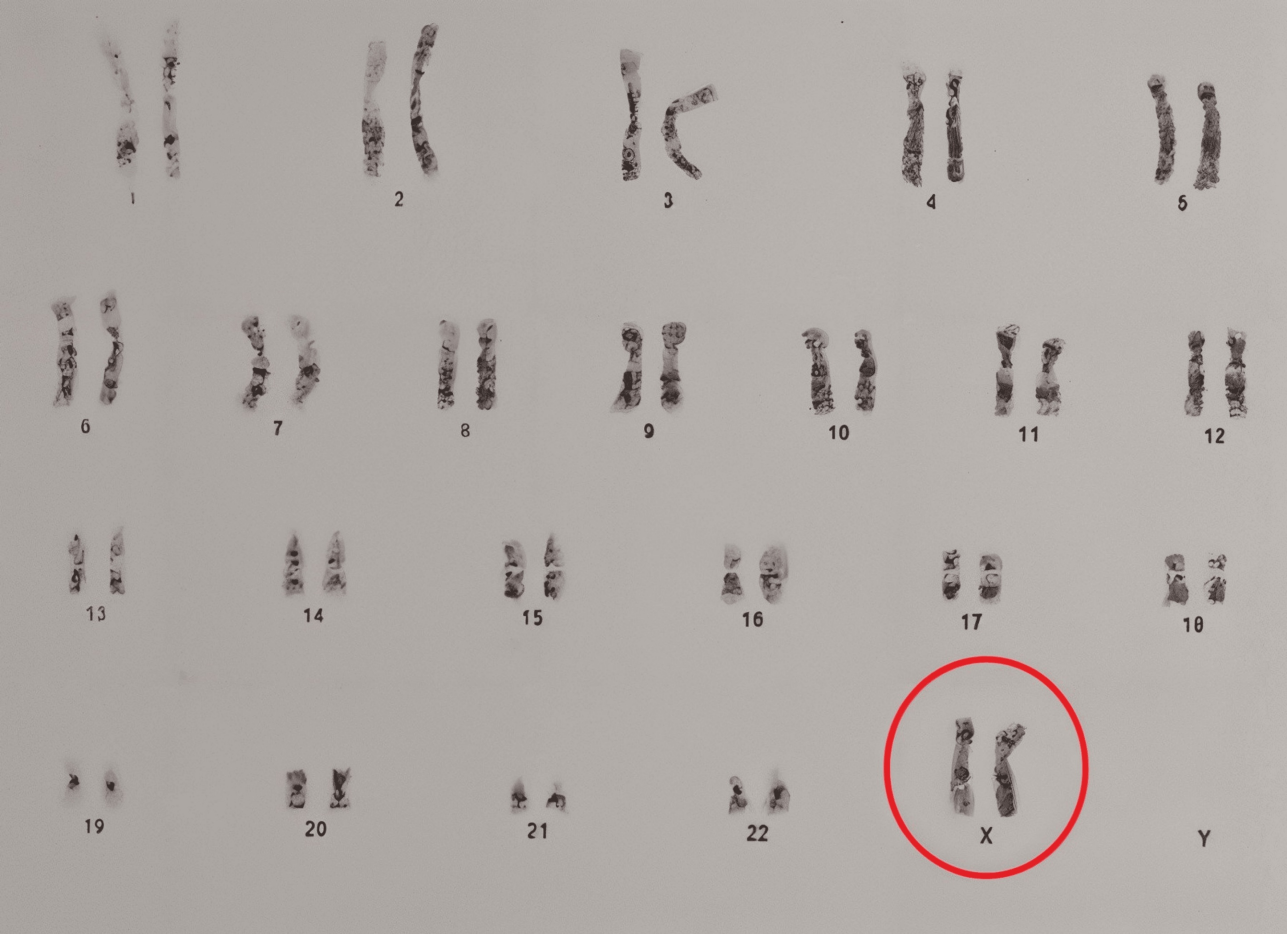
Peripheral chromosomal analysis shows a normal female karyotype, 46,XX. The red circle indicates the normal female sex chromosomes XX.

Due to the presence of streak ovaries, endo-ovarian failure, and FSH in the menopausal range, she was started on oral contraceptive pills (OCP) containing Ethinyl estradiol in combination with Levonorgestrel based on a working diagnosis of Turner syndrome. However, she did not exhibit any other Turner syndrome features, and her height did not meet the criteria for the condition. She was then suspected to have Turner Mosaic Syndrome and was kept on OCPs. Swyer syndrome was also considered. At the age of 22, a subsequent cytogenetic study of her peripheral blood revealed a normal karyotype 46XX, excluding all the previously mentioned syndromes and shrouding the case in mystery (Figure 4).

The patient remained undiagnosed and continued on oral contraceptive pills for six months, which initiated menstruation and led to minimal breast growth. However, due to the contraceptive pills, she also experienced side effects such as mood swings and depression, which led to her discontinuing the medication and later taking them inconsistently. Resuming the drug would result in normal bleeding, but stopping it would result in amenorrhea. She then stopped using OCPs entirely and experienced complete amenorrhea.

At the age of 26, after consulting another doctor and based on her karyotype, gonadal dysgenesis, and hearing loss, she was diagnosed with PRLTS. However, due to limited resources, no molecular analysis could be conducted to identify the gene involved in the mutation.

It is considered that providing this patient with hormones will help her develop her secondary sexual characteristics and her infantile uterus, increasing her chances of becoming fertile through ovum donation. However, due to financial constraints and religious objections, this treatment option is still up for debate, necessitating more research in the field of the uncommon PRLTS.

## Discussion

Literature review

The various presentations and complications of PRLTS reported in the literature have been summarized in Table 2.

**Table 2:** Past reports of Perrault syndrome (PRLTS).

Author	Age of Diagnosis	Chief Complaint	Ovarian Dysfunction	Neurological Abnormalities	Radiological or Clinical Findings	Molecular Analysis/ Genetic Testing	Treatment
Carminho-Rodrigues MT et al. [4]	8 years	Bilateral severe hearing loss	Undetected	Absent	Pelvic ultrasound: normal prepubertal uterus measuring 32 mm. A right ovary measuring 16 × 6 × 6 × 6.5 mm was visualized. The left ovary was not clearly visualized. Hormonal assessments: normal prepubertal values of follicle-stimulating hormone (FSH) (2.8 IU/L) and luteinizing hormone (LH) (0.7 U/L).	Two missense variants in the LARS2 gene	Cochlear implant
Sampathkumar G et al. [5]	18 years	No menarche, deaf, mute	Poorly developed breasts (Tanner stage 2), sparse pubic and axillary hair (Tanner stage 2)	Absent	Ultrasonogram of abdomen and pelvis showed hypoplastic uterus and streak ovaries.	N/A	Replacement hormonal therapy initially with estrogen and later combined with progesterone
Munson HE et al. [6]	4.5 years	Abnormal gait	FSH level was 15.8 mIU/ml	Unsteady gait, speech regression, bilateral deep tendon reflexes absent at ankles and knees, sensory ataxia, hearing loss	Ultrasound of the pelvis was significant for an absence of ovarian structures.	Heterozygous state with two variants in the TWNK gene	Cochlear implant
Pan Z et al. [1]	23 years	Hearing loss	Oligomenorrhea with a hormone profile indicative of increased gonadotropin levels	Absent	Reduced ovarian follicles and multiple Naboth cysts on the cervix on ultrasound.	Compound heterozygous missense variants in the LARS2 gene	Hearing aids
Roberts LM and Carnivale B [7]	20 years	Amenorrhea, hearing loss	Elevated FSH (145.4) and LH Tanner III breast and pubic hair development	Absent	Small uterus, measuring 4.1x1.4x2.2cm, and streak ovaries, with a small amount of fluid in the endometrial canal and a stripe of 2mm. Right ovary was 0.7x1.1x1cm and left ovary was 0.6x1x0.6cm.	Not performed	Oral Contraceptive Pills

Discussion

We describe a case of a girl diagnosed with PRLTS after undergoing several misdiagnoses, including Turner syndrome and Mosaic Turner syndrome. PRLTS results from several genetic mutations including CLPP, TWNK, HARS2, HSD17B4, or LARS2 [3]. The proteins produced by these genes play a vital role in mitochondrial function. Research has speculated that disruption of energy production by mitochondria could explain the clinical manifestations of PRLTS [8]. The various presentations and complications of Perrault syndrome reported in the literature have been summarized in Table 2.

The patient in our case report first consulted for her condition at the age of 17 years when she presented with the chief complaint of primary amenorrhea and failure to develop secondary sexual characteristics. Initially, the doctors suspected Turner syndrome. Associated with the complete loss of one X chromosome in all cells, common manifestations of Turner syndrome include short stature, cardiac abnormalities, late puberty, fertility issues, hypergonadotropic hypogonadism, ovarian dysgenesis, endocrine, and autoimmune disorders [9]. In this case, small atrophied ovaries indicating ovarian failure also matched the misdiagnosis. However, due to her tall stature (174 cm) and the absence of other symptoms commonly found in Turner as mentioned above, this diagnosis was ruled out. Nevertheless, differential diagnoses should include Turner syndrome, as half of Turner patients have a degree of hearing loss.

The doctors then considered another possibility: Turner mosaic, defined as the partial loss of X chromosomes in a few cells and presenting with milder phenotypic abnormalities compared to classic Turner syndrome, with the most common karyotypes being 45X/46XX [10]. Additionally, they also considered the possibility of a male karyotype due to the infantile uterus and streak ovaries presented on ultrasound, hence considering Swyer syndrome. Individuals with a female phenotype and female external genital organs who have a 46,XY karyotype and streak gonads that should be removed due to their high risk for malignancy are said to have pure gonadal dysgenesis, also known as Swyer syndrome [11]. However, the doctor could not reach a definitive conclusion, and the patient continued on oral contraceptive pills for another year until her karyotype testing was done, revealing a normal karyotype, 46XX. This further deepened the diagnostic uncertainty.

She was complaining of sensorineural hearing loss, which was gradually worsening. In PRLTS, bilateral hearing loss presents ranging from moderate to profound in individuals [4]. The age of onset varies from 18 months to the age of 32 years. Furthermore, neurological symptoms such as gait ataxia, pyramidal dysfunction, behavioral problems, and neurodevelopmental delay are present, which, however, did not present in this patient [12]. van der Knaap MS et al. discussed three cases who developed neurological features with high severity later in life [13]. Our patient, however, did not experience any radiological or clinical neurological abnormalities. Assessment of hearing loss should be done and managed by a multidisciplinary team, which includes an otolaryngologist and an audiologist. Interventions for patients with hearing loss encompass hearing aids, cochlear implantation, and vibro-tactile devices, which can be options for children above 12 months with progressive hearing loss [4]. In patients with PRLTS, sensorineural deafness is regarded as part of the widespread neurological involvement. PRLTS is classified into two types, type I, known as static, which has absent neurological features, and type II, with progressive neurological disease [5]. Our patient most likely developed type I of PRLTS as she did not present with any neurological manifestations.

As per our report, the patient did not start menstruating, which confirmed amenorrhea, and she also did not develop secondary sexual characteristics appropriate for her age. Commonly, only TWNK-related PRLTS presents with gonadal dysfunction. Therefore, it is suggested that children presenting with clinical signs of ataxia and polyneuropathy should be evaluated for Perrault Syndrome, specifically when gonadal dysfunction is present in females [6]. Additionally, attention should be given to the presence of sensorineural hearing loss when making differential diagnoses.

For the treatment of ovarian insufficiency, patients with primary amenorrhea in adolescence can benefit from hormonal replacement therapy to induce puberty [1]. This patient was started on oral contraceptive pills, which induced her menstruation and led to minimal development of secondary sexual characteristics, such as breasts. However, females suffering from gonadal dysgenesis can opt for assisted reproductive techniques through ovum donation. Additionally, oocyte cryopreservation can be considered for females at risk of primary ovarian insufficiency [1].

Previous research has shown a predominance of females in PRLTS, although the mode of inheritance is autosomal recessive. The manifestations of ovarian dysfunction are resulting in the increased recognition of PRLTS in females in addition to hearing loss and ataxia [6]. In females, the average age of a confirmed diagnosis is 22 years, following a history of deafness associated with delayed puberty [5]. The patient in this case presented for consultation with amenorrhea being the chief complaint. The hallmark of PRLTS in females is ovarian dysgenesis, and sensorineural deafness is present in both males and females. The karyotype, however, is typically normal for affected individuals [5].

The diagnosis of PRLTS can be challenging due to the similar presentations of other more common syndromes. Thus, karyotyping, molecular analysis, and genetic testing to identify the mutated genes must be performed in such cases before establishing a definitive diagnosis.

## Conclusions

In conclusion, this case underscores the challenges and complexity in diagnosing PRLTS, a rare genetic disorder characterized by ovarian dysfunction and sensorineural hearing loss in females. However, PRLTS may not be rare but might be unrecognized and misdiagnosed. The patient, initially misdiagnosed with Turner syndrome and Mosaic Turner syndrome due to overlapping clinical features, ultimately presented with a normal 46,XX karyotype, steering the diagnosis towards PRLTS. This case highlights the importance of thorough karyotyping, molecular analysis, and genetic testing in differentiating PRLTS from other conditions with similar presentations. Furthermore, the lack of neurological symptoms in this patient suggests a diagnosis of type I PRLTS, which is marked by the absence of progressive neurological features, although most cases reported until now have some degree of neurological deficit. The patient’s response to hormonal therapy, which induced menstruation and minimal breast development, indicates the potential for such interventions in managing ovarian insufficiency in PRLTS. However, the decision to pursue fertility treatments remains complex, influenced by ethical and financial considerations. This case reinforces the need for a multidisciplinary approach and further research to enhance the understanding and management of PRLTS.
